# Dr. Thomas Harris

**Published:** 1861-05

**Authors:** 


### NECROLOGICAL NOTICES.

DR. THOMAS HARRIS expired in this city on the 4th of March, at the age of seventy-eight; having been, at the time of his death, the oldest surgeon in the American navy. For several years he had been at the head of the Medical Bureau at Washington. He was a graduate of the University of Pennsylvania in the days of Rush, Dorsey, Wistar, Barton, and Physick. His private practice in this city was for a long time very large, respectable, and lucrative. He was associated for a number of years with Chapman's Philadelphia Institute as lecturer on surgery, was a good operator, and contributed some valuable papers to the medical periodical press, and to Dr. Hays's American Cyclopædia of Practical Medicine and Surgery. Urbane and affectionate in his manners, he was universally popular, and greatly esteemed by his professional brethren. He was twice married. His eldest son is a surgeon in the navy. The immediate cause of his death was apoplexy, of which he had had one or two attacks several years previously.

